# A highly efficient organogenesis protocol based on zeatin riboside for in vitro regeneration of eggplant

**DOI:** 10.1186/s12870-019-2215-y

**Published:** 2020-01-06

**Authors:** Edgar García-Fortea, Agustín Lluch-Ruiz, Benito José Pineda-Chaza, Ana García-Pérez, Juan Pablo Bracho-Gil, Mariola Plazas, Pietro Gramazio, Santiago Vilanova, Vicente Moreno, Jaime Prohens

**Affiliations:** 1 Instituto Universitario de Conservación y Mejora de la Agrodiversidad Valenciana, Universitat Politècnica de València, Camí de Vera s/n, 46022 Valencia, Spain; 2 Instituto de Biología Molecular y Celular de Plantas, Consejo Superior de Investigaciones Científicas-Universitat Politècnica de València, 46022 Valencia, Spain; 3 Faculty of Life and Environmental Sciences, University of Tsukuba, 1-1-1 Tennodai, Tsukuba, 305-8572 Japan

**Keywords:** Zeatin riboside, Regeneration, Somatic organogenesis, *Solanum melongena*, Tetraploids

## Abstract

**Background:**

Efficient organogenesis induction in eggplant (*Solanum melongena* L.) is required for multiple in vitro culture applications. In this work, we aimed at developing a universal protocol for efficient in vitro regeneration of eggplant mainly based on the use of zeatin riboside (ZR). We evaluated the effect of seven combinations of ZR with indoleacetic acid (IAA) for organogenic regeneration in five genetically diverse *S. melongena* and one *S. insanum* L. accessions using two photoperiod conditions. In addition, the effect of six different concentrations of indolebutyric acid (IBA) in order to promote rooting was assessed to facilitate subsequent acclimatization of plants. The ploidy level of regenerated plants was studied.

**Results:**

In a first experiment with accessions MEL1 and MEL3, significant (*p* < 0.05) differences were observed for the four factors evaluated for organogenesis from cotyledon, hypocotyl and leaf explants, with the best results obtained (9 and 11 shoots for MEL1 and MEL3, respectively) using cotyledon tissue, 16 h light / 8 h dark photoperiod conditions, and medium E6 (2 mg/L of ZR and 0 mg/L of IAA). The best combination of conditions was tested in the other four accessions and confirmed its high regeneration efficiency per explant when using both cotyledon and hypocotyl tissues. The best rooting media was R2 (1 mg/L IBA). The analysis of ploidy level revealed that between 25 and 50% of the regenerated plantlets were tetraploid.

**Conclusions:**

An efficient protocol for organogenesis of both cultivated and wild accessions of eggplant, based on the use of ZR, is proposed. The universal protocol developed may be useful for fostering in vitro culture applications in eggplant requiring regeneration of plants and, in addition, allows developing tetraploid plants without the need of antimitotic chemicals.

## Background

Common eggplant (*Solanum melongena* L.), also known as brinjal eggplant, is one of the most important vegetables, globally ranking fifth in total production among vegetable crops [1]. In addition, this crop has a great interest due to its high content in bioactive compounds, mostly phenolics, that have multiple properties beneficial for human health [2]. In vitro culture has been of great relevance for the genetic improvement of this crop, including the development of doubled haploids to obtain pure lines [3], or the development of the first commercial transgenic *Bt* eggplant [4]. However, as it occurs in other crops such as onion [5–7] or gerbera [8], available protocols to regenerate eggplants are mostly inefficient or highly dependent on the genotype [9–11]. Thus, more efficient and reproducible protocols suitable to a wide range of genotypes are needed to circumvent the current drawbacks for in vitro regeneration in eggplant, mainly those related to the strong effect that the genotype has on regeneration efficiency, and even more if we take into account that globally over 6600 eggplant accessions are currently available in the germplasm Genesys database [12].

The development of new genome editing technologies in plant breeding has generated a growing interest in in vitro culture for regeneration protocols [13, 14]. This is partially due to the difficulties often encountered in plant regeneration, which is a key step in any transformation protocol and a major bottleneck for applying these techniques in many species [13, 15]. On the other hand, other breeding techniques, like the development of polyploids without antimitotic products such as colchicine [16–19], can benefit from regeneration protocols able to induce a certain percentage of plants with changes in ploidy levels. Tetraploids can also be the starting point for obtaining triploids which, in addition to the lack of seeds, can exhibit superior quality parameters in the fruit.

Zeatin riboside (ZR; C_15_H_21_N_5_O_5_), is a cytokinin of interest for several plant science applications. Since its discovery by Letham [20] in immature corn kernels, it has revealed as a useful plant growth regulator in a wide range of crops, particularly since the 1990s. One of the earliest applications of ZR for in vitro culture was for protoplasts regeneration in several species, such as *Brassica nigra* (L.) W.D.J. Koch [21], *Vigna sublobata* Roxb [22]., or *Solanum lycopersicum* L. [23]. ZR was also used for regeneration from leaf explants, such as in potato (*Solanum tuberosum* L.), where ZR was the cytokinin that resulted in the greater number of shoots per explant [24]. In addition, ZR was used for somatic embryogenesis induction from cotyledon protoplasts, such as in tomato [25], and for shoots induction in axillary buds of bracts in plants from genera *Aloe*, *Gasteria*, and *Haworthia* [26]. More recently, ZR has been used to circumvent the generally low percentage of seed germination in the African baobab (*Adansonia digitate* L.), where the efficient micropropagation from axillary buds in a medium supplemented with ZR has allowed the efficient propagation of plants of this species [27]. In the olive tree (*Olea europaea* L.), ZR has been successfully used for micropropagation from nodal segments, replacing the methodology of hardwood cutting, which is a very time-consuming technique [28]. These reports reveal that ZR has proved to be a very effective plant growth regulator on different in vitro culture applications in several species.

Two studies suggested that ZR may improve the organogenic regeneration efficiency in eggplant. Singh [29] proposed a transformation method of plastids in eggplant by using ZR in the culture medium. Muktadir [30] evaluated the capacity of organogenesis induction in five varieties of eggplant by comparing the effect of different growth regulators, obtaining the best results using an MS medium supplemented with ZR and IAA. More recently, ZR was also used in the production of eggplant doubled haploids from anthers using a modified medium proposed by Rotino [31] by adding 1 mg/L of ZR and 3 mg/L of naphthaleneacetic acid (NAA) [32].

Here, we evaluate the effects of ZR at different concentrations and in combination with IAA on different plant tissues (cotyledon, hypocotyl and leaf) of genetically and phenotypically diverse eggplant accessions under different light conditions (photoperiod and dark). In order to obtain highly reliable estimates of the traits and parameters studied, around 4300 explants were evaluated. Our study is aimed at providing relevant information on the ZR effects in in vitro regeneration in eggplant. As a result, we developed an efficient regeneration protocol that is not only useful for genetically and phenotypically distinct eggplant accessions, but also for a related wild species.

## Results

### Explant type, induction conditions, accession, and culture media effects in eggplant regeneration

**Fig. 1:**
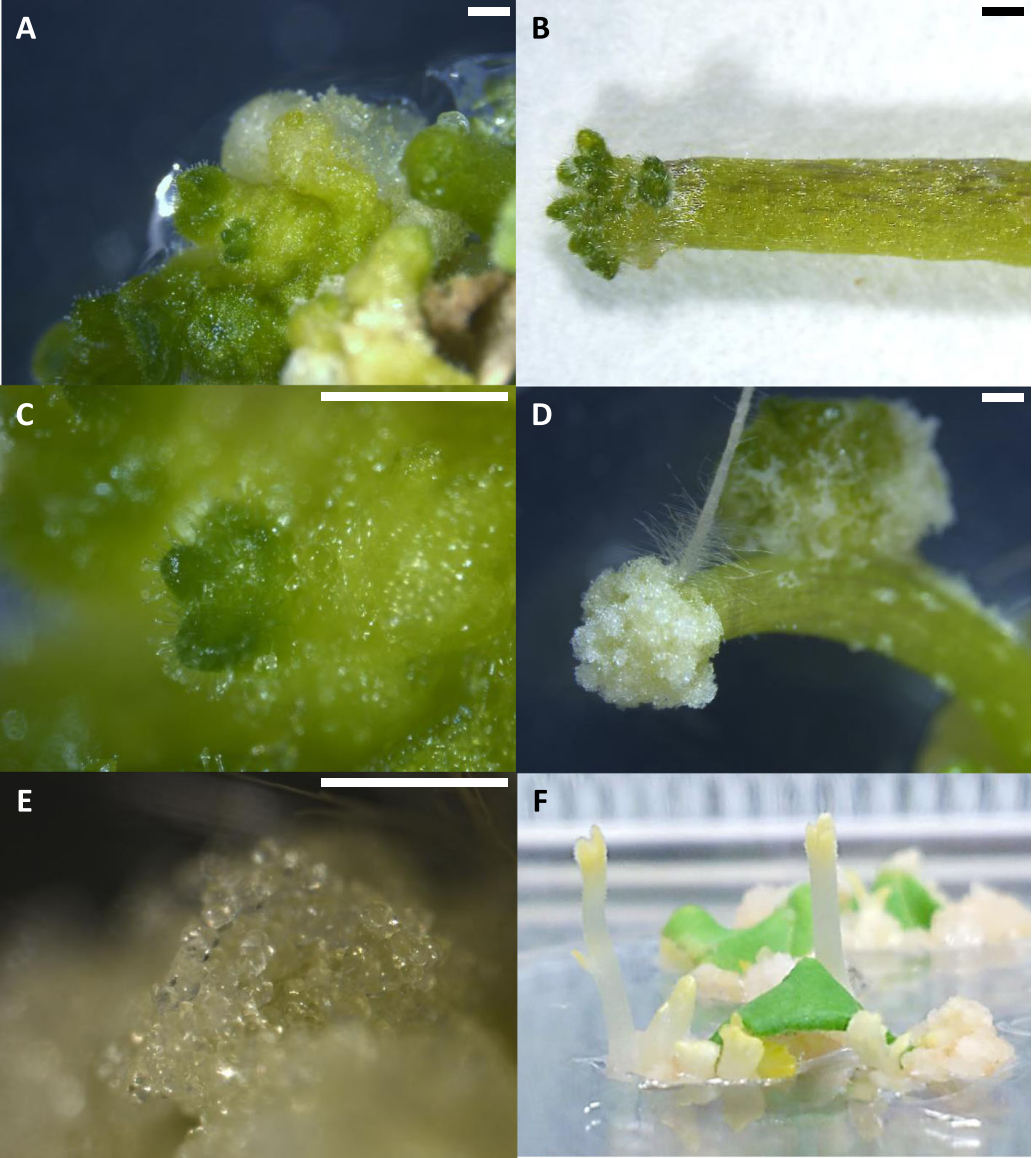
Initiation of bud formation in cotyledonary tissue of eggplant under 16 h light / 8 h dark photoperiod culture conditions (**a**); formation of shoots in hypocotyl tissue (**b**); organized structure and the formation of the first trichomes in cotyledonary tissue (**c**); callus formed in hypocotyl tissue under light culture conditions (**d**); which, at further magnification can be observed as a disorganized cell structure (**e**); appearance of the buds formed in cotyledonary tissue in 24 h dark photoperiod culture conditions, with elongated growth and absence of chlorophyll in the apex being observed (**f**). All the images were taken after a month of culture. The size of the bars is 1 mm

The formation of non-organogenic friable calli was an event that, although observed in all the proven culture media, did not occur in abundance. Nevertheless, meristematic nodes were observed on the surface of the compact organogenic calli of cotyledon tissues (Fig. 1a) and at one edge of the hypocotyl tissues (Fig. 1b). These structures had an organized appearance, green colour and in most cases presented trichomes on the surface (Fig. 1c). On the other hand, non-organogenic friable calli were observed both in cotyledons and at the edge of some hypocotyl explants (Fig. 1d). These structures consisted of transparent and disorganized cells that disintegrated easily when touched with tweezers (Fig. 1e). A relevant morphological event was the shoots formation after a month in dark culture conditions (Fig. 1f). These had an elongated growth habit and presented a pale coloration that turned green shortly after being transferred into the light.

In the experiment 1, significant differences (*P* < 0.05) were observed for the main effects of tissue and culture medium in the regeneration of shoots, calli and roots, and for accession in the induction of calli (Table 1). Overall, the tissue potentially more organogenic for shoot regeneration was the cotyledon with an average of 2.55 shoots/explant, followed by the hypocotyl with 1.66 shoots/explant, and the leaf with 0.70 shoots/explant. Although wide ranges of variation were observed for shoot formation, their median and mode values ranged between 0 and 2 and between 0 and 1, respectively, indicating that most explants produced a limited number of shoots. Hypocotyl explants produced more calli, on average almost 1.5-fold than cotyledons or leaves, while for root regeneration cotyledon was the one with the highest average number of adventitious roots generated (0.51 per explant) followed by hypocotyl (0.31) and leaf (0.24), however, no significant differences were observed for root formation between the two induction conditions (photoperiod and darkness). Regarding the accession effect, the only differences observed in regeneration were for the formation of calli, which were higher in MEL1 than in MEL3 (Table 1). The incubation in light conditions after a month favoured the appearance of shoots, with an average of 1.67 shoots/explant, while incubation in the dark gave lower values, of 1.07 explants/shoot. Light had no significant effects on the development of other types of organs. Many significant differences were observed among culture media for shoots regeneration, roots and calli production (Table 1). The media with higher average values for shoot formation were E4 and E5, with over 2.2 shoots/explant, followed by E6 and E3 (2.04 and 1.54 shoots/explant). On the other hand, media E1, E7 and the control E0 had the lowest regeneration rate for shoots, with averages between three and four-fold lower than the best media. All the media tested displayed a formation of calli higher than the control medium (E0) while those without ZR and containing IAA (media E1 and E7) promoted the formation of friable non-organogenic calli (Fig. 1d and e). Finally, the E1 and E7 media resulted in the highest rate of root formation, much higher than the control medium E0, while the rest of media gave lower ratios than E0 (Table 1). Regarding the percentage of buds of shoots, 100% of the explants cultured in media containing ZR (E2-E6) formed shoots; however, explants grown in the media E0, E1 and E7 barely formed shoots (0–3%). Similarly, the percentage of explants with callus for the E2-E6 media, as well as for E1 medium (2 mg/L IAA), was 100%. Again the E0 (control) medium and E7 medium (0.1 mg/L IAA) barely presented 1–5% explants with callus.The combination of cotyledon explants and light induction was the one that gave the best performance in shoots formation. For these reason the data subsets corresponding to the combination of these two treatments were studied for the best media (E3 to E6) for the two accessions (MEL1 and MEL3) (Table 2). The analysis of hypocotyl explants data was repeated following the same approach (Table 3). By using these combinations, we observed a clear positive interaction for the number of shoots, with means higher than those expected for the sum of the main effects. In the case of cotyledon explants, the range (0–26) for shoots formation remained similar, however, the median and the mode increased their values, indicating that in general this combination of factors increased the number of shoots obtained. For hypocotyls, the changes were not as dramatic as in the case of cotyledons. The mean for shoots formation increased slightly while the range decreased considerably, the mode and the median were stable with a value around 2. This shows that hypocotyls under these conditions produce a limited number of shoots, significantly lower than those of cotyledons. However, for both cotyledon and hypocotyl, the values ​​of mean, median, mode and range for the formation of non-organogenic calli and of roots were very low. The mode in the case of hypocotyls had a value of two, showing that callus formation in this type of tissue is more frequent than in cotyledon.

**Table 1:** Mean, median, mode, and range for the organs produced during organogenesis in experiment 1 for each of the different levels for the four factors evaluated. Three experimental sessions with three replicates for each combination of factors and five explants per Petri dish were used

Factors	Shoots				Calli				Roots			
	Mean^a^	Median	Mode	Range	Mean^a^	Median	Mode	Range	Mean^a^	Median	Mode	Range
Tissue												
Cotyledon	2.55 c	0	0	0–26	1.13 a	1	0	0–4	0.51 c	0	0	0–4
Hypocotyl	1.66 b	1	1	0–14	1.52 b	2	2	0–2	0.31 b	0	0	0–2
Leaf	0.70 a	0	0	0–13	1.09 a	1	0	0–4	0.24 a	0	0	0–4
Accession												
MEL1	1.35 a	0	0	0–20	1.16 a	1	2	0–4	0.30 a	0	0	0–4
MEL3	1.41 a	0	0	0–26	1.41 b	2	2	0–4	0.32 a	0	0	0–4
Induction condition												
Light/Dark (12/8 h)	1.67 b	1	0	0–26	1.28 a	1	2	0–4	0.31 a	0	0	0–4
Dark (24 h)	1.07 a	0	0	0–15	1.29 a	1	2	0–4	0.30 a	0	0	0–4
Medium												
E0	0.45 a	0	0	0–3	0.44 a	0	0	0–2	0.24 c	0	0	0–2
E1	0.39 a	0	0	0–5	1.18 c	1	2	0–3	0.80 d	1	0	0–4
E2	1.10 b	0	0	0–13	1.62 e	2	2	0–4	0.14 a	0	0	0–2
E3	1.54 c	1	0	0–14	1.47 e	2	2	0–4	0.23 b	0	0	0–4
E4	2.21 d	1	0	0–22	1.54 e	2	2	0–4	0.15 a	0	0	0–4
E5	2.54 d	2	0	0–21	1.57 e	2	2	0–4	0.09 a	0	0	0–2
E6	2.04 c	1	0	0–26	1.32 d	1	2	0–4	0.12 a	0	0	0–4
E7	0.56 a	0	0	0–5	0.85 b	1	0	0–3	0.75 d	1	0	0–3

^a^For each factor, means separated by different letters are significantly different at *p* < 0.05 according to the non-parametric pairwise Wilcoxon test

**Table 2:** Mean, median, mode, and range for the organs produced during organogenesis in experiment 1 for explants grown from cotyledon incubated under 16 light / 8 h dark photoperiod conditions for the levels of media E3 to E6 in accessions MEL1 and MEL3. Three experimental sessions with three replicates for each combination of factors and five explants per Petri dish were used

Medium	Shoots				Calli				Roots			
	Mean^a^	Median	Mode	Range	Mean^a^	Median	Mode	Range	Mean^a^	Median	Mode	Range
MEL1												
E3	6.7 a	5	14	0–14	1.4 a	1	3	0–3	0.1 a	0	0	0–1
E4	8.3 a	6	3	0–20	2.1 a	2	1	0–4	0.4 a	0	0	0–1
E5	7.8 a	7	2	2–17	1.8 a	1.5	3	0–3	0.1 a	0	0	0–1
E6	9 a	9	9	2–19	1.4 a	1.5	0	0–3	0 a	0	0	0
MEL3												
E3	7.6 a	4	4	0–17	1.7 a	1	1	1–3	0.1 a	0	0	0–1
E4	10.1 a	4	3	0–22	1.6 a	1	1	0–3	0.1 a	0	0	0–1
E5	7.8 a	5	4	0–21	2 a	2	3	1–3	0.3 a	0	0	0–1
E6	11 a	4	4	0–26	1.6 a	1	3	0–3	0.2 a	0	0	0–1

^a^For each combination of accession (MEL1 or MEL3) and organogenic structure (shoots, calli, or roots), medium means separated by different letters are significantly different at *p* < 0.05 according to the non-parametric pairwise Wilcoxon test

**Table 3:** Mean, median, mode, and range for the organs produced during organogenesis in experiment 1 for explants grown from hypocotyl incubated under 16 light / 8 h dark photoperiod conditions for the levels of media E3 to E6 in accessions MEL1 and MEL3. Three experimental sessions with three replicates for each combination of factors and five explants per Petri dish were used

Medium	Shoots				Calli				Roots			
	Mean^a^	Median	Mode	Range	Mean^a^	Median	Mode	Range	Mean^a^	Median	Mode	Range
MEL1												
E3	2.3 a	2	2	0–8	1.8 a	2	2	1–2	0.1 a	0	0	0–2
E4	2.1 a	2	2	0–6	1.7 a	2	2	1–2	0.1 a	0	0	0–1
E5	2.9 a	2	1	0–9	1.6 a	2	2	1–2	0.2 a	0	0	0–1
E6	2.1 a	2	2	0–7	1.5 a	2	2	0–2	0.2 a	0	0	0–1
MEL3												
E3	2.7 a	2	2	0–7	1.7 a	2	2	1–2	0.1 a	0	0	0–1
E4	2.5 a	2	2	0–7	1.8 a	2	2	1–2	0.0 a	0	0	0–1
E5	3.5 a	3	2	0–8	1.6 a	2	2	1–2	0.0 a	0	0	0–1
E6	2.5 a	2	2	0–6	1.7 a	2	2	0–2	0.1 a	0	0	0–1

^a^For each combination of accession (MEL1 or MEL3) and organogenic structure (shoots, calli, or roots), medium means separated by different letters are significantly different at *p* < 0.05 according to the non-parametric pairwise Wilcoxon test

No significant differences for cotyledon (Table 2) or for hypocotyl (Table 3) explants were observed among the four culture media when evaluating all the effects together under these selected conditions. For both genotypes no significant differences were observed among media for cotyledon and hypocotyl, although the average of the E6 medium for the formation of shoots was higher for cotyledon explants in both genotypes. Given that medium E6 presented a higher average in shoots development in the case of cotyledon tissue and being the less expensive medium, it was chosen for the regeneration protocol.

### Comparison of media for rooting of shoot explants

**Fig. 2:**
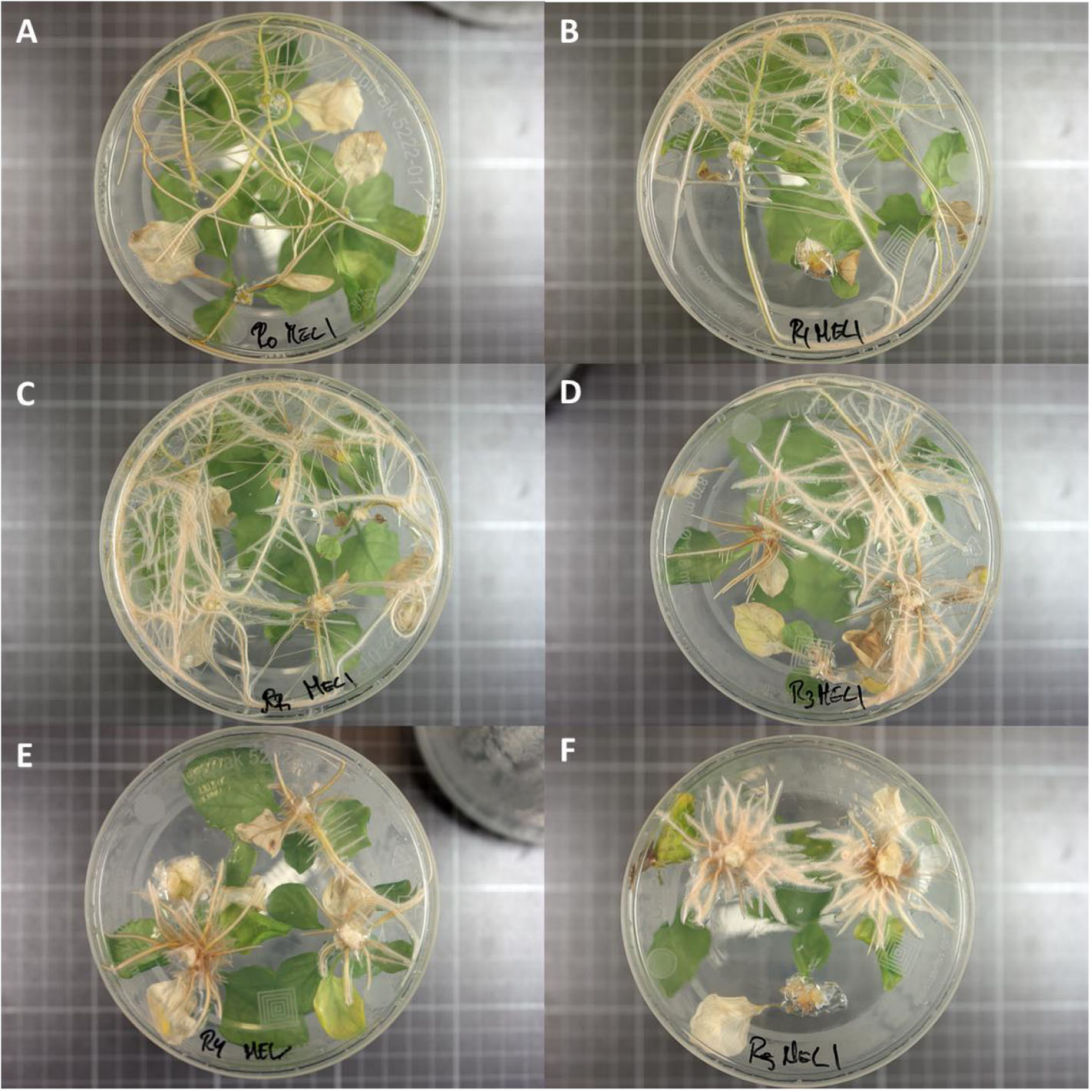
Response in the in vitro formation of roots in common eggplant accession MEL1 in response to different concentrations of IBA in media E0 (0 mg/L; **a**), R1 (0.5 mg/L; **b**), R2 (1 mg/L; **c**), R3 (2 mg/L; **d**), R4 (3 mg/L; **e**) and R5 (4 mg/L; **f**)

The rooting performance of MEL1 shoot explants for the six culture media was evaluated in the experiment 2. An increase of IBA concentration resulted in a greater formation of main roots (media R3, R4 and R5), a decrease in the number of secondary roots, as well as a greater thickness and a reduction in the length of the main root (Table 4; Fig. 2). Medium E0 (without IBA) presented the lower average of root formation per shoots, while the highest, with 7.6 main roots/shoot, was obtained in medium R5 (4 mg/L of IBA). On the other hand, the number of secondary roots reached its maximum value (3.8) in the medium R2 (1 mg/L of IBA). For concentrations of 2 mg/L of IBA (medium R3) and higher, the number of secondary roots decreased reaching its lowest value in medium R5 with 0.8 secondary roots per explant. The thickness of the main root varied between 1.1 to 2.6 mm, increasing with higher IBA concentrations in the medium, while the length varied between 1.1 to 2.5 cm decreasing when the concentration of IBA increased.

**Table 4:** Mean, median, mode, and range for the main roots, secondary roots and thickness and length of the main root per plant produced during rooting of shoot explants in experiment 2 in the accession of common eggplant MEL1. Three experimental sessions with three replicates and five explants per Petri dish for each medium were used

Medium	Main Roots				Secondary Roots				Main Roots thickness^b^				Main Roots length^c^			
	Mean^a^	Median	Mode	Range	Mean^a^	Median	Mode	Range	Mean^a^	Median	Mode	Range	Mean^a^	Median	Mode	Range
E0	1.3 a	0	1	0–4	3.2 b	0	1	0–10	1.1 a	1	1	1–2	2.5 c	3	3	1–3
R1	3.2 b	3	3	0–7	3.7 c	0	3	0–10	1.2 a	1	1	1–2	2.6 c	3	3	2–3
R2	4.8 bc	2	4	0–10	3.8 c	0	3	0–10	1.4 ab	1	1	1–2	2.5 c	3	3	1–3
R3	5.3 c	10	5.5	0–10	1.6 ab	0	0	0–8	1.8 b	2	2	1–3	1.7 b	2	2	1–2
R4	5.9 c	0	7	0–10	1.4 ab	0	0	0–10	2.0 b	2	2	1–3	1.2 a	1	1	1–2
R5	7.6 c	0	8.5	0–18	0.8 a	0	0	0–10	2.6 c	3	0	1–3	1.1 a	1	1	1–2

^a^For each trait, medium means separated by different letters are significantly different at *p* < 0.05 according to the non-parametric pairwise Wilcoxon test

^b^Measured in a scale (1 = < 0.8 mm; 2 = 0.8 to 1 mm; 3= > 1 mm)

^c^Measured in a scale (1 = < 3 cm; 2 = 3 to 4.5 cm; 3= > 4.5 cm)

### Validating the regeneration protocol in different genotypes

After choosing the best conditions and medium, the protocol developed was tested in other genetically diverse materials of common eggplant [IVIA371, black beauty (BB), MM1597] and in INS1. As hypocotyls are also available when germinating the seeds for obtaining cotyledons, their regeneration capacity was also evaluated. The number of shoots regenerated was high for all genotypes, although significant differences among accessions were observed in the production of shoots per explant both for cotyledons and hypocotyls (Table 5). For cotyledons, IVIA371, BB, and INS1 displayed similar means (around seven shoots/explant), while MM1597 had the lowest average (3.1 shoots/explant). In the case of hypocotyls, IVIA371 displayed the highest number of shoots, with an average of 6.5 shoots/explant, while BB presented the lowest shoots number (1.8 shoots/explant).

**Table 5:** Mean, median, mode, and range for the organs produced during organogenesis in experiment 3 for explants grown from cotyledon and hypocotyl incubated under 16 light / 8 h dark photoperiod conditions and using medium E6 in accessions of common eggplant IVIA371, BB, and MM1597 and INS1. Three experimental sessions with three replicates for each combination of factors and five explants per Petri dish were used

Accessions	Shoots				Calli				Roots			
	Mean^a^	Median	Mode	Range	Mean^a^	Median	Mode	Range	Mean^a^	Median	Mode	Range
Cotyledon												
IVIA371	6.8 b	7	7	0–12	1.8 b	2	2	0–2	0.1 b	0	0	0–1
BB	6.7 b	6	3	2–13	1.9 b	2	2	1–2	0.1 b	0	0	0–1
MM1597	3.1 a	3	0	0–13	1.2 a	1	2	0–2	0.1 b	0	0	0–1
INS1	7.4 b	7.5	5	1–13	1.8 b	2	2	1–2	0 a	0	0	0–0
Hypocotyl												
IVIA371	6.5 c	7	6	0–16	1.5 a	2	2	0–1	0.2 a	0	0	0–2
BB	1.8 a	1	0	0–6	1.6 ab	2	2	1–2	0.3 a	0	0	0–3
MM1597	4.4 b	4	6	0–11	1.5 a	2	2	0–2	0.6 a	0	0	0–2
INS1	5.8 bc	5.5	9	0–15	1.7 b	2	2	0–2	0.1 a	0	0	0–1

^a^For each explant type (cotyledon or hypocotyl), medium means for each trait separated by different letters are significantly different at *p* < 0.05 according to the non-parametric pairwise Wilcoxon test

**Fig. 3:**
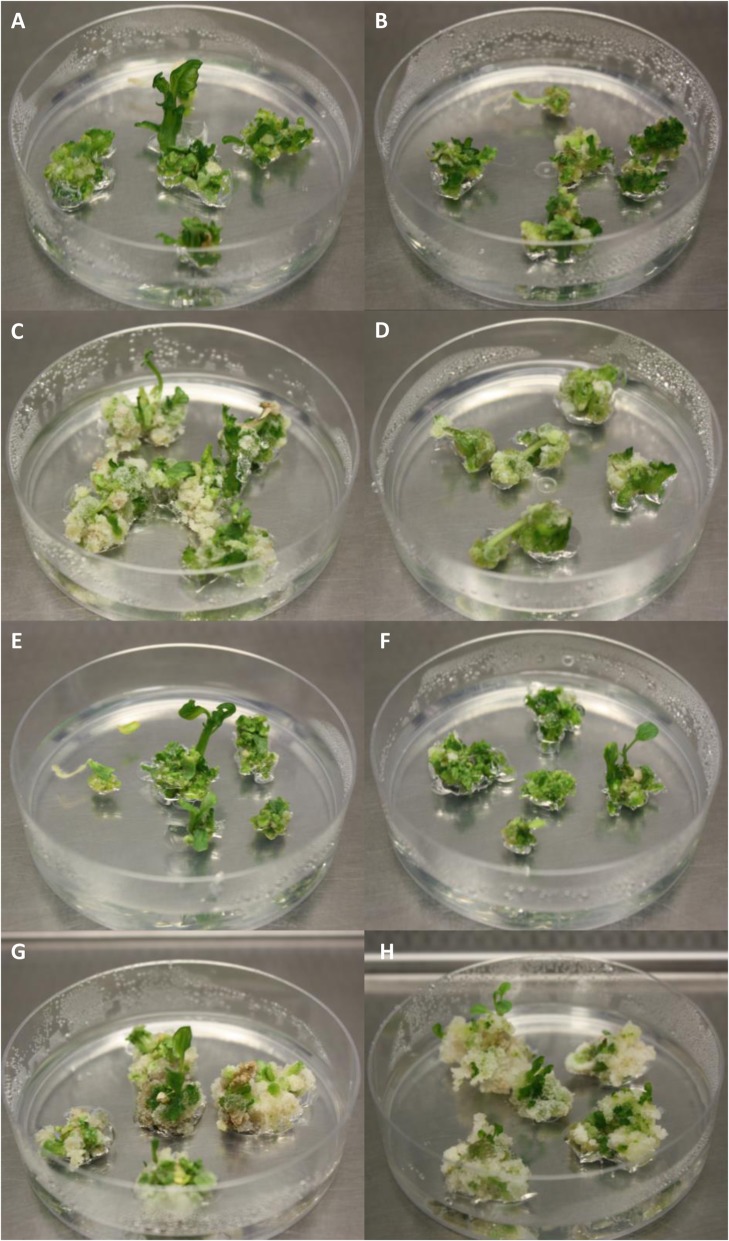
Organogenic response of the different eggplant accessions for the E6 medium cultivated under light conditions for cotyledons (left) and hypocotyls (right) for common eggplant accessions IVIA371 (**a** and **b**), BB (**c** and **d**), and MM1597 (**e** and **f**), and for INS1 (**g** and **h**). Differences were observed between the two tissues, being the cotyledon the tissue with the best average results in all the cases, except for MM1597. Small differences between the accessions were also observed, like the larger size and the non-friable aspect of INS1 callus (**g** and **h**)

IVIA371 and MM1597 formed completely compact organogenic calli (Fig. 3a-b and e-f) while for BB and INS1 most of the calli were friable and non-organogenic (Fig. 3c-d and g-h). This occurred for both cotyledon and hypocotyl tissues. For callus formation, INS1 (Fig. 3g and h) gave the highest number of calli in hypocotyl, with values significantly higher than those of IVIA371 and MM1597 (Table 5). The formation of roots in this medium was very limited and, in most explants, no adventitious roots were formed (Table 5).

### Acclimatizated plants with the selected protocol for regeneration and rooting

**Fig. 4:**
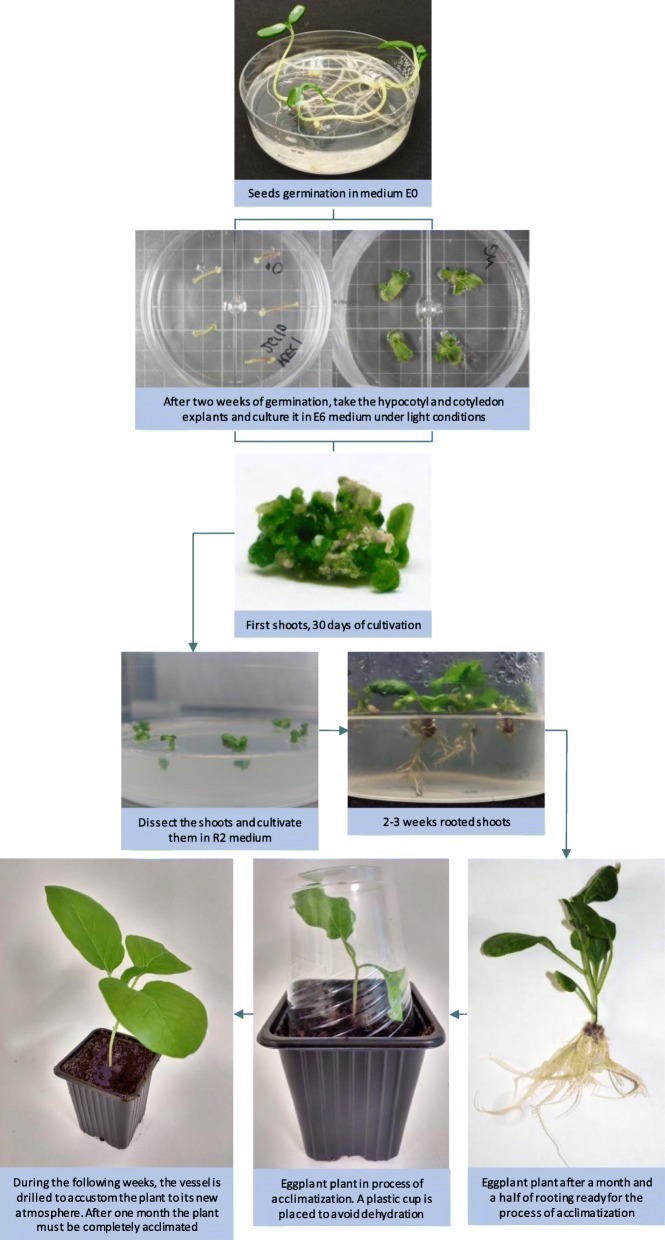
Proposed universal protocol for the regeneration of eggplant plants from cotyledon and hypocotyl. Starting from tissue of seeds sterilized and cultivated in vitro in medium E0, the combination of medium E6 and light conditions for both hypocotyl and cotyledon tissue is proposed. Subsequently, after approximately 1 month of cultivation, the shoots are placed in rooting medium R2 until their root system is ready for the transplant and the process of acclimatization

Table 6 summarizes the results of the number of explants with shoots, acclimated plants, and plants per initial explant obtained for the different accessions cultured in vitro using the selected protocol for in vitro regeneration of eggplant, which consists of using light conditions, the E6 medium for the induction of shoots, and the medium R2 for in vitro root induction (Fig. 4). By using this universal protocol, all the accessions showed very high yields with percentages of explants with shoots close to or greater than 70% in all cases. The percentage of acclimatizated plants by initial explant ranged between 28.88 and 80.00% for cotyledon explants and between 20.00 and 46.76% for hypocotyl explants.

**Table 6:** Percentage (±SE) of explants with shoots and number of acclimatized plants from the experiment 3 for the accessions of common eggplant IVIA371, BB, and MM1597, and for *S. insanum* INS1 in two different tissues (cotyledon and hypocotyl) using 16 h light / 8 h dark photoperiod conditions and the E6 medium for organogenesis and the medium R2 for root formation induction. The percentage (±SE) of the diploid (2x) and tetraploid (4x) regenerated plants in experiment 4 are also reported. The number of initial explants used for each accession was *n* = 45

Accessions	Explants with shoots (%)	Acclimatized plants	Acclimatized plants / initial explants (%)	2x regenerants (%)	4x regenerants (%)
Cotyledon					
IVIA371	96.66 ± 0.03	36	80.00 ± 0.06	50.00 ± 0.08	50.00 ± 0.08
BB	100.00 ± 0.00	15	33.33 ± 0.07	61.30 ± 0.13	38.70 ± 0.13
MM1597	69.23 ± 0.07	13	28.88 ± 0.06	64.71 ± 0.13	35.29 ± 0.13
INS1	100.00 ± 0.00	24	53.33 ± 0.07	75.00 ± 0.09	25.00 ± 0.09
Hypocotyl					
IVIA371	86.66 ± 0.06	13	28.88 ± 0.06	65.00 ± 0.13	35.00 ± 0.13
BB	70.00 ± 0.08	21	46.76 ± 0.07	66.70 ± 0.10	33.30 ± 0.10
MM1597	86.66 ± 0.06	9	20.00 ± 0.06	76.92 ± 0.14	23.08 ± 0.14
INS1	90.00 ± 0.05	17	37.77 ± 0.07	74.80 ± 0.11	25.20 ± 0.11

### Ploidy level analysis

**Fig. 5:**
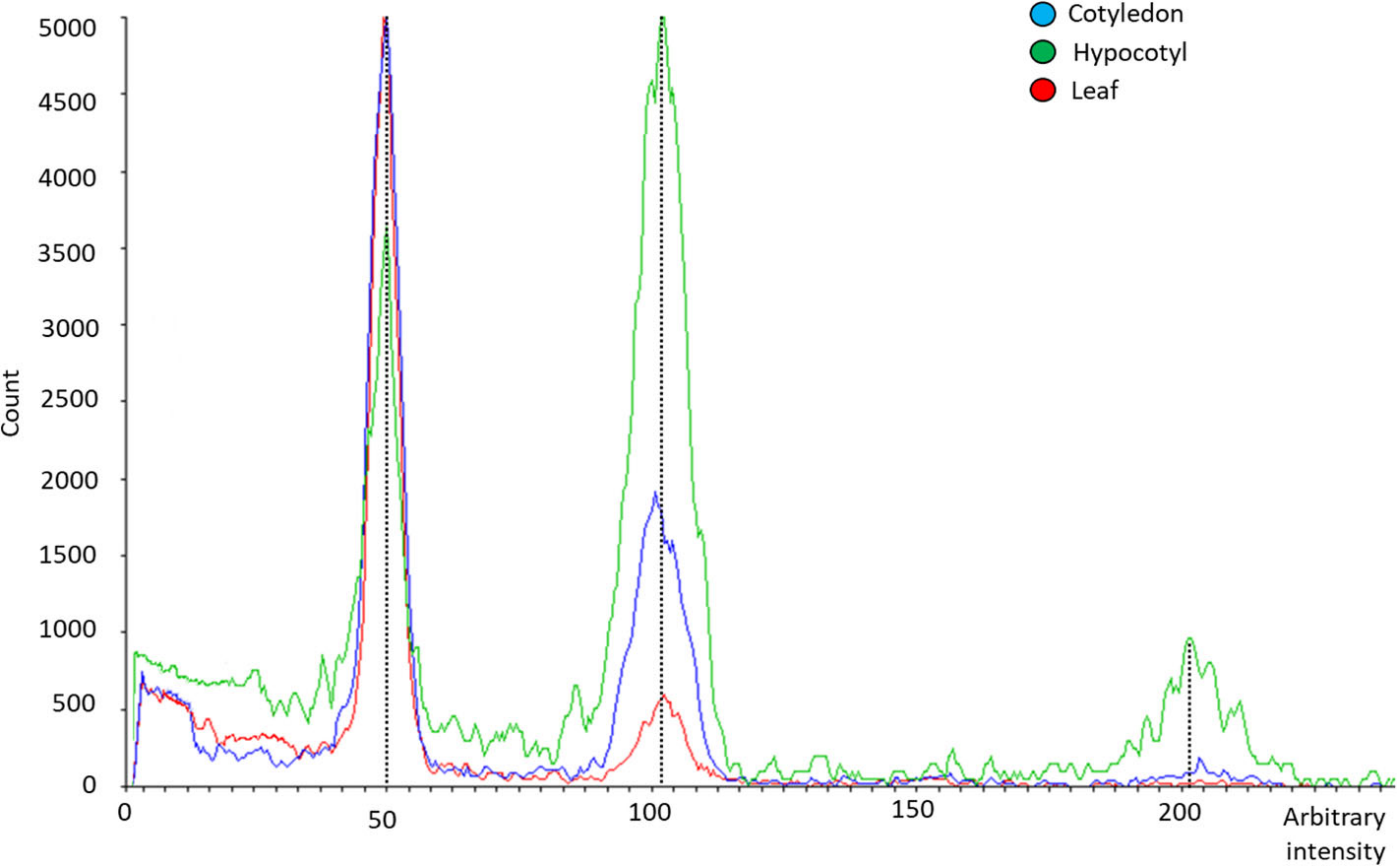
Flow cytometry histogram of the relative nuclear DNA contents of different tissues from eggplant accession IVIA371: cotyledon (blue), hypocotyl (green) and leaf (red). The x-axis represents the proportional fluorescence intensity level to the nuclear DNA quantity. The different polysomatic profiles of the different tissues analyzed can be observed. The peak located at the value 50 correspond to the diploid nuclei in phase G1, the peak located at the value 100 corresponds to the sum of the diploid nuclei in phase G2 and the tetraploid nuclei in phase G1, while the one at the value 200 represents tetraploid nuclei in G2 phase. The y-axis indicates the number of nuclei analyzed

A similar polysomatic pattern was observed in the different accesions when cotyledon, hypocotyl and leaf tissue explants were analized by flow cytometry (Fig. 5). The cotyledon had between three and five times more cells than the leaf at G2 phase peak. In the case of hypocotyl, the number of cells of the G2 peak was between seven and nine times greater than the peak of the leaf sample. Both the cotyledon and hypocotyl tissues showed a peak at the value of 200 arbitrary units of fluorescence, corresponding to tetraploid cells in division, while in the case of the leaf, no such peak was observed (Fig. 5).

In addition, the ploidy level of regenerated plants was also evaluated. The results revealed that between 25 and 50% of the regenerated plants were tetraploids (Table 6). The percentage of polyploid plants was similar for both cotyledon and hypocotyl as well as among accessions, except for cotyledons of IVIA371 where 50% of the regenerated plants were tetraploid.

## Discussion

The development of regeneration methods to obtain plants in vitro is essential for many applications in micropropagation and plant breeding. To our knowledge, up to now there are no universal methods for the regeneration of eggplant. An example of this is the plethora of works providing different conditions, growth regulators and media for eggplant regeneration, but none of them provides a universal and reproducible protocol in a diverse group of genetically diverse accessions. Some examples are the protocols based on the use of thidiazuron (TDZ) [33], NAA or in combination with benzylaminopurine (BAP) [34, 35], or BAP in combination with IAA [36]. Even explants irradiated with helium-neon laser were evaluated for organogenesis in eggplant [37]. However, all these protocols were highly genotype dependent.

ZR is a plant growth regulator that has been successfully used for many crops and plant species like *Brassica nigra* [21], *Solanum lycopersicum* [23] or *Olea europaea* [28], although it has been much less used than other cytokinins. In this study, we developed a new universal regeneration protocol based on the use of ZR that has proven to be highly efficient in eggplant, which is a genotype dependent recalcitrant species to in vitro culture [9–11]. Due to the good results showed by ZR in some crops including two seminal reports on eggplant [29, 30], we aimed at developing a universal protocol for regeneration of eggplant based on the use of ZR. Thus, in our study, we tested genotypes representative of eggplant diversity [38], including an accession of the wild ancestor INS1 [39], using a large number of combinations of factors and replicates.

Cotyledon was the most organogenic tissue for all conditions, giving better results under 16 h light / 8 h dark than in the 24 h dark photoperiod conditions. Similar results were observed in another study in which the organogenic capacity of the cotyledon and hypocotyl tissues in the eggplant was evaluated [36]. This organogenic capacity of the cotyledons has also been reported in other crops such as melon [40], tomato [41], or peanut [42]. Regarding the composition for the culture media, no significant increases in the response were observed for concentrations over 2 mg/L of ZR. In other studies in eggplant, the concentrations determined to be optimal for ZR were 1 mg/L in the case of plastid transformation [29] and anthers [32], and 2 mg/L in the case of hypocotyl culture [30]. These results are in agreement with those obtained in our study. In other crops, the optimum concentration of ZR for the induction of organogenesis were variable, being 0.8 mg/L for potato [24], 1 mg/L for tomato protoplasts [22], 1.89 mg/L in the case of some succulent plants [26], and much higher in woody plants such as olive tree where the optimum concentration was set in 4.77 mg/L [28]. In general, concentrations higher than 2 mg/L resulted in a decrease of the organogenic response, as in hypocotyl protoplasts of *Vigna sublobata* [22] or tomato cotyledons [25], where ZR in combination with gibberellic acid (GA_3_) and IAA did not show inductive effect on the tissues. Other works confirmed that ZR presented lower regeneration results when it is used in combination with auxins. For example, Rolli [27] reported a decrease in the percentage of regeneration when ZR was used in combination with IBA for African baobab. Similar effects were also observed for brassicas where 2 mg/L of ZR promoted the shoot formation from organogenic calli, but when ZR was combined with 0.2 mg/L of IAA, the shoot induction effect was suppressed, resulting in the formation of somatic embryogenesis [21]. For IAA, negative effects were observed at concentrations higher than 0.5 mg/L [25]. Our results are in agreement with these studies, since the best media found in our study (E6) lacked IAA.

When comparing the results of the E6 medium with the rest of the media we observed a higher average number of outbreaks per explant than the rest of the media. Medium E6 also showed higher percentages of organogenic regeneration compared to the medium proposed by Muktadir [30] where ZR (2 mg/L) was used in combination with 0.1 mg/L of IAA. This is further evidence that IAA does not contribute, or even decrease the organogenesis induction in eggplant.

The rooting medium R2 (1 mg/L IBA) displayed the highest yield in the formation of secondary roots, providing a greater number of root nodes for functional roots ex vitro. In fact, although the roots formed in vitro are not generally functional ex vitro [43, 44], they may serve as scaffolds for the new functional secondary roots that can be formed from their nodes. We also observed that an increase in the concentration of IBA higher than 2 mg/L increased the thickness of the roots, reduced their length and promoted the formation of the main roots while reducing the formation of the secondary roots. This is not desirable for the subsequent acclimatization of the plants due to the low number of nodes formed by the absence of secondary root [43]. In this experiment the roots formed without problems under photoperiod conditions, this would reinforce the results observed in experiment 1 where we did not see differences in the formation of adventitious roots between the induction conditions evaluated (photoperiod and darkness) suggesting that the light does not condition the in vitro root formation in the case of this crop.

Despite the genetic divergence between MEL1 and MEL3 [38], we did not detect significant differences in the organogenic response between these two accessions. In the rest of accessions, including one of the wild ancestor of eggplant INS1 [39], the response of cotyledons was very similar, whereas, in the case of hypocotyls, small differences between genotypes were observed. These results indicate that the genotype effect on the regeneration efficiency in this protocol is low and it can applicable to a wide range of genotypes, including eggplant wild relatives. Although many shoots per explant were formed with our protocol, the number of acclimated plants per explant was much lower. This is because not all shoots were large enough to subculture them in the rooting media. This step might be easyly improved by incorporating an elongation step prior to the rooting. For example, explants with shoots could be subcultured in an growth regulator-free E0 medium [11, 45], or in a medium with a concentration of 1.5 mg/L GA_3_ [3]. In this sense it is worth mentioning that the cultivation of small shoots during one or 2 weeks in continuous darkness promotes their elongation without the weak etiolation harming the normal future development of the shoots in photoperiod conditions (data not shown).

All accessions had a very similar general polysomatic profile, in which cotyledons and hypocotyls showed higher rates of initial polyploid cells than those of the leaf. This is probably due to the fact that the first endoreplications occurred in the cotyledon and hypocotyl during germination, while the events of polysomatia in the rest of tissues occur at later stage [16, 17]. Although cotyledons had a percentage of polyploid cells lower than the hypocotyl, the proportion of polyploid plants regenerated from both tissues was similar. This may be due to the greater organogenic capacity of the cotyledons that we report in this work. A significant proportion of the regenerated plants were stable tetraploids, which indicated that it is a highly efficient method for the development of tetraploids without the need to use chemical antimitotic agents such as colchicine. Similar results have been observed in different studies such as in in vitro culture of tomato hypocotyls where 42.3% of the plants were tetraploid [46] or in culture of protocorm-like bodies in *Phalaenopsis* species, where an average polyploid production was obtained between 36.0–74.9% depending on the species [47].

## Conclusions

We developed a universal protocol for the eggplant regeneration. This universal protocol consists in the cultivation of cotyledons in a medium (E6) with ZR at 2 mg/L under light conditions for 1 month, and afterwards rooting them in medium R2, which contains IBA at 1 mg/L. Taking advantage of the polysomaty displayed by cotyledon and hypocotyl tissues, this protocol can also be used to obtain stable non-chimeric polyploid plants without the need to use antimitotic agents. This protocol, based on the use of ZR, and the rest of the information provided in this study can foster in vitro breeding, transformation and genetic editing of eggplant being an excellent base to start working. It may be necessary to adapt the protocol depending on the selective agents used in the transformation process. For this, it would be convenient to perform toxicity tests of different concentrations of antibiotics or other selective agents and assess whether the regeneration capacity of the transformed cells is compromised or not. Our approach, in which ZR proved of great value for developing a highly efficient regeneration protocol in an otherwise recalcitrant species, might be of interest in other related recalcitrant crops.

## Methods

### Plant material

**Fig. 6:**
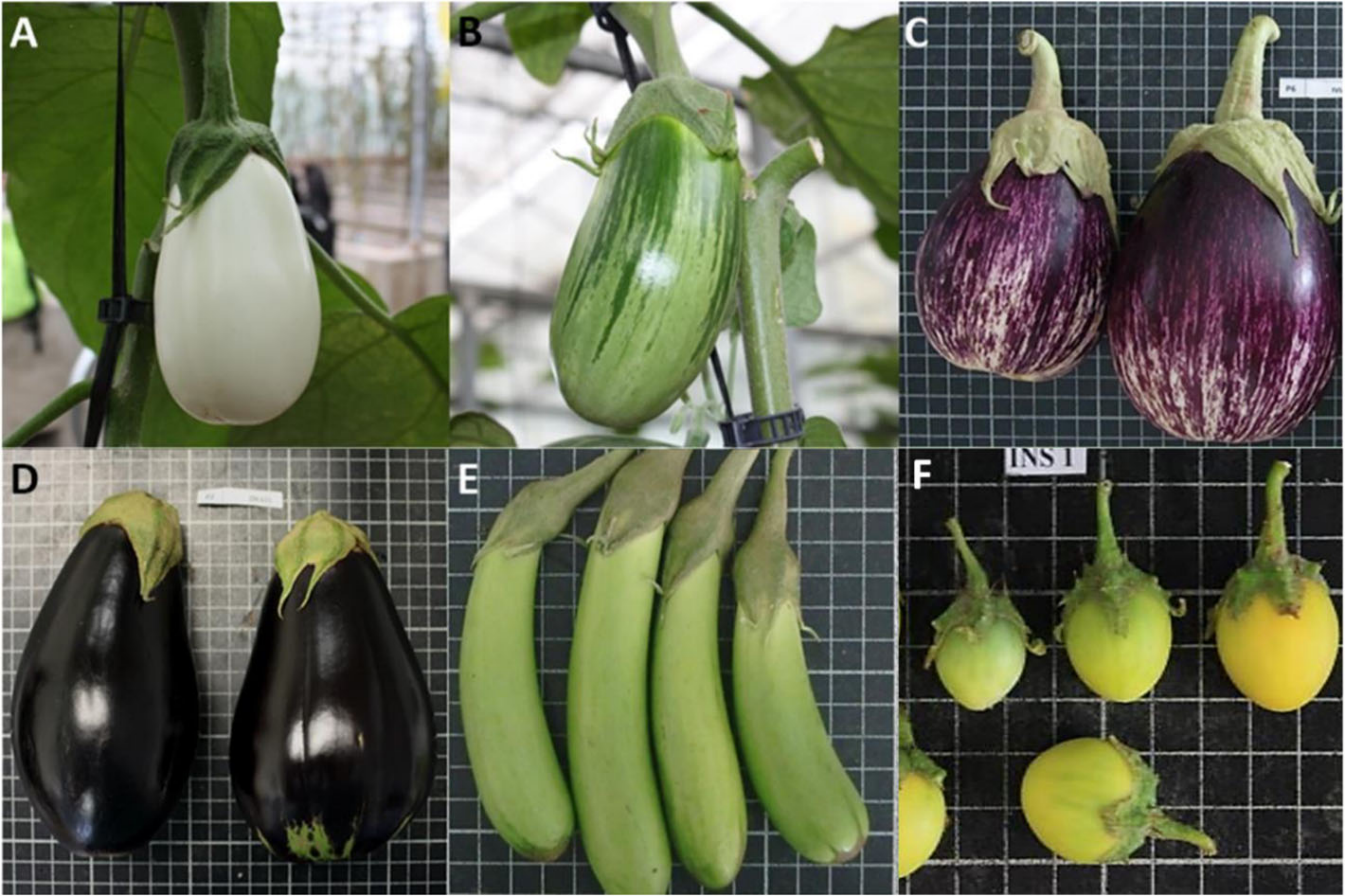
Fruits of the common eggplant (*S. melongena*) accessions [MEL 1 (**a**); MEL3 (**b**); IVIA371(**c**); BB (**d**) MM1597 (**e**)] and of the wild ancestor *S. insanum* [INS1 (**f**)] used in the different experiments, revealing the high phenotypic diversity of the materials studied. The size of the grid cells is 1 × 1 cm

Five *S. melongena* accessions, namely MEL1, MEL3, IVIA371, BB, and MM1597, and one of *S. insanum* (INS1), the wild ancestor of eggplant and the only wild species in the primary genepool of eggplant [39, 48], were used (Fig. 6). Seeds of the six accessions were kindly provided by the germplasm bank of Universitat Politècnica de València (Valencia, Spain; FAO germplasm bank code: ESP026). These materials have different origins (Ivory Coast for MEL1 and MEL3, Spain for IVIA371, Italy for BB, India for MM1597, and Japan for INS1), have been previously identified taxonomically, and characterized morphologically and genetically, displaying a wide variation for morphological and agronomic traits, as well as for genetic diversity [38, 49, 50]. MEL1 and MEL3, bearing white and green fruits respectively, are the recipient parents of backcross programmes for the development of introgression lines with wild relatives, and have been used in several breeding programmes [51–53].

### Experimental layout and workflow

**Fig. 7:**
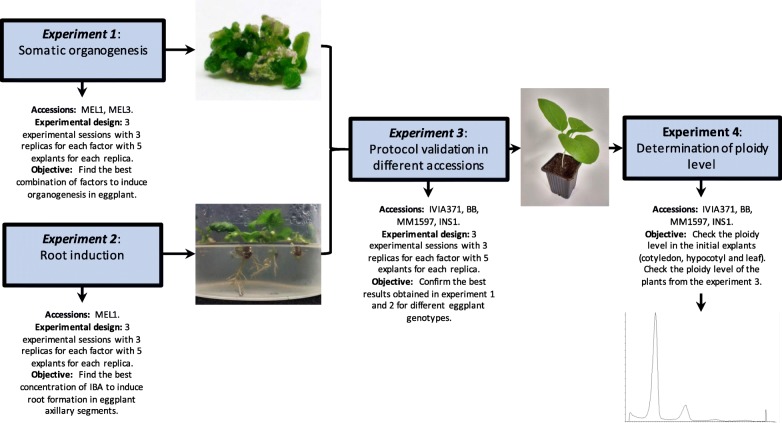
Description of the accessions, the experimental design and the main objectives of the four experiments performed. The flow of the arrows indicates the order in which the experiments were conducted and their relationships

The experimental layout and workflow are presented in Fig. 7. The first experiment was aimed at evaluating the effect of different factors in somatic organogenesis in eggplant. Cotyledon, hypocotyl and leaf tissue from MEL1 and MEL3 were tested in eight culture media with different concentrations of ZR and IAA combined with two induction conditions (light and dark). In parallel, for the second experiment, six culture media were evaluated to determine which of them stimulates more efficiently root induction. This step was carried out only with MEL1. After running the first two experiments and having established the best protocols among those tested for the eggplant regeneration, in the third experiment these were tested in the other four accessions (IVIA371, BB, MM1597 and INS1) in order to assess the genotype effect. Finally, in the fourth experiment, the ploidy of the plants regenerated from this protocol was evaluated. Due to the detection of polyploid plants, the polysomatic pattern of cotyledon, hypocotyl and leaf tissues was also checked by flow cytometry.

### Growth conditions of the starting material

For in vitro germination, seeds were previously surface-sterilized by immersion in 70% ethanol for 30 s, followed by 10 min in a solution of 5% w/v sodium hypochlorite with 0.1% (v/v) of Tween 20, and finally washed three times with sterilized distilled water. After sterilization, seeds were germinated in the darkness on solid medium E0 (Table 1). The medium E0 was distributed in Petri dishes of 9 cm diameter and also in plastic pots of 9.7 cm diameter and 11 cm height fitted with a membrane filter in the lid to allow gaseous exchange (Microbox containers O118/120 + OD118/120 #10 (G), SAC02, Nevele, Belgium). The plastic pots were used for growing the plantlets to obtain leaf explants. To synchronize the explants types used in the experiment (cotyledon, hypocotyl and leaf), firstly 50 seeds of each of the MEL1 and MEL3 accessions were sown in plastic pots to obtain leaves. Two weeks later other 50 seeds of each of these two accessions were sown in Petri dishes and kept under dark culture conditions, to obtain the cotyledons and hypocotyls. The plastic pots were placed in a climatic chamber, at a temperature of 25 °C with a photoperiod of 16 h light / 8 h darkness. At the same time, 50 additional MEL1 seeds were sown in plastic pots with E0 medium for the rooting protocol experiment (Table 7). They were kept in the same climatic chamber for 4 weeks under the same conditions.

**Table 7:** Zeatin riboside (ZR) and indolacetic acid (IAA) concentrations of the different induction media for the in vitro germination of seeds (E0) and somatic organogenesis of eggplant explants (E0 to E7)

Growth regulator	Medium							
	E0	E1	E2	E3	E4	E5	E6	E7
ZR (mg/L)	0	0	1	2	3	4	2	0
IAA (mg/L)	0	2	0.5	0.1	0.05	0	0	0.1

### Experiment 1: somatic organogenesis

Explants were cultured on different organogenic induction media containing ½ MS basal salts [54], 1,5% (w/v) sucrose, 0,7% (w/v) gelrite, and supplemented with one of seven different growth regulator combinations (Zr, 0–4 mg/L; IAA, 0–2 mg/L) (Table 7). From seedlings grown in Petri dishes in dark conditions, the proximal and distal parts of the cotyledons were excised while the hypocotyls were cut into fragments of about 1 cm long. The leaves of the plantlets grown in plastic pots were cut into fragments of one square centimetre. Fifteen explants for each of the three tissues (cotyledon, hypocotyl or leaf) were divided into three plates (five explants per Petri dish). This was triplicated for each of the organogenesis media conditions; thus, 846 Petri dishes and 4320 explants (3 plant tissues × 2 accessions × 8 inductions media × 2 incubation (light/dark) conditions × 3 plates per plant tissue × 3 experimental replicas) were evaluated.

Half of the plates of each combination of medium and type of explant were kept in light conditions (100–112 μmol m^− 2^ S^− 1^) in the culture chamber at 25 °C with 16/8 h light/dark photoperiod. The other half were kept in dark conditions in the same culture chamber, by wrapping the plates with aluminium foil. After 1 month, the number of shoots, calli and roots of each explant were counted, and the plates that were in dark conditions were transferred to light conditions.

### Experiment 2: root induction

For developing a rooting induction protocol of the explants regenerated with the different combinations of ZR (0–4 mg/L) and IAA (0–2 mg/L), different IBA concentrations (0–4 mg/L) were used on a medium containing ½ MS basal salts, 1.5% (w/v) sucrose, 0.7% (w/v) gelrite (Table 8). Five explants with axillary buds from MEL1 were planted per plastic box. The plastic pots were taken to the culture chamber and placed at 25 °C and 16 h / 8 h of light/dark photoperiod. After 1 month, the number of primary and secondary roots was counted and the length and thickness (diameter) of the primary roots were evaluated. This was triplicated for each of the rooting media conditions; thus, 54 Petri dishes and 270 explants (1 accession × 6 inductions mediums × 3 plastic pots × 3 experimental replicas) were evaluated.

**Table 8:** Indolbutyric acid (IBA) concentrations of different rooting media evaluated in explants from internodal sections from MEL1 eggplant accession

Growth regulator	Medium					
	E0	R1	R2	R3	R4	R5
IBA (mg/L)	0	0.5	1	2	3	4

### Experiment 3: protocol validation in different accessions

The common eggplant accessions IVIA371, BB, MM1597 and the INS1 accession were used for this third experiment. The same seed disinfection protocol mentioned above for experiment 1 was used, and 50 seeds of each of the four accessions were sown in Petri dishes of 9 cm diameter with medium E0. The plates were brought to the culture room under the same temperature conditions used in the former experiments (25 °C) in dark conditions for 2 weeks.

The proximal and distal parts of the cotyledons were excised, and the hypocotyls were cut into fragments of about 1 centimetre. For each accession, fifteen cotyledons and hypocotyls explants were distributed over three plates (five per plate per type of explant), with E6 medium, in three experimental sessions, totalling 1440 explants evaluated in this experiment. The plates were maintained into the culture chamber under light conditions, at 25 °C and a photoperiod of 16 h/ 8 h light/dark. After 1 month, the number of shoots, roots and calli of each individual explant was counted.

### Experiment 4: determination of ploidy level

Cell nuclei from different tissues (cotyledon, hypocotyl and leaf) were isolated mechanically according to Dpooležel [55]) with modifications. Cotyledon, hypocotyl, and leaf sections of approximately 0.5 cm^2^ were chopped with a razor blade in a 6 cm diameter glass Petri dish containing 0.5 ml lysis buffer LB01 (pH 7.5) supplemented with 15 mM Tris (hydroxymethyl) aminomethane, 2 mM Na_2_EDTA and 0.5 mM spermine, and incubated for 5 min. Subsequently, the suspension containing nuclei and cell fragments was filtered using a 30 μm CellTrics filter (Sysmex, Sant Just Desvern, Spain). The nuclei in the filtrate were stained with CyStain UV Ploidy (Sysmex) and incubated for 5 min. The fluorescence intensity of the homogenate was measured using a CyFlow ploidy-analyzer (Partec, Münster, Germany), measuring at least 4000 nuclei for each sample. Using young leaves of a diploid eggplant, the diploid control peak was established at 50 points of the arbitrary intensity value of the fluorescence in the histogram (Fig. 5). By comparison with this peak, the ploidy of the other tissues evaluated was checked. The ploidy of cotyledons, hypocotyls and leaves was evaluated to verify their polysomatic pattern. For experiment 3, also young leaves from regenerated plants were evaluated.

### Statistical analysis

Independence among variables (distribution-plot test), homoscedasticity (Bartlett’s test), and normality (Shapiro-Wilk test) were evaluated for the experiments 1, 2 and 3. Given that for none of these experiments the three criteria were met, the Kruskal-Wallis non-parametric test followed by the pairwise Wilcoxon test at *p* < 0.05 was used to evaluate statistical significance of differences. All these analyses were carried out using R software [56]. The mean, median and the mode of each factors were calculated to complete the information in the data sets. For the data from the experiment 4, the relative percentage for the ploidy levels each genotype along with its standard error was calculated.

## Data Availability

The datasets used and/or analysed and plant materials used in the current study are available from the corresponding author on reasonable request.

## Abbreviations

BAP: Benzylaminopurine
GA_3_: Gibberellic acid;
IAA: Indoleacetic acid
IBA: Indolebutyric acid
NAA: Naphthalene acid
TDZ: Thidiazuron
ZR: Zeatin riboside

## References

[1] FAO (2019). FAOSTAT Food and Agriculture.

[2] Gürbüza N, Uluişikb S, Frarya A, Frary A, Doğanlar S (2018). Health benefits and bioactive compounds of eggplant. Food Chem.

[3] Rivas-Sendra A, Corral-Martínez P, Camacho-Fernández C, Seguí-Simarro JM (2015). Improved regeneration of eggplant doubled haploids from microspore-derived calli through organogenesis. Plant Cell Tissue Organ Cult.

[4] Shelton AM, Hossain MJ, Paranjape V, Azad AK, Rahman ML, Khan ASMMR, Prodhan MZH, Rashid MA, Majumder R, Hossain MA, Hussain SS, Huesing JE, McCandless L (2018). Bt eggplant project in Bangladesh: history, present status, and future direction. Front Bioeng Biotechnol.

[5] Muren RC (1989). Haploid plant induction from unpollinated ovaries in onion. Hortscience.

[6] Campion B, Bohanec B, Javornik B (1995). Gynogenic lines of onion (*Allium cepa* L.): evidence of their homozygosity. Theor Appl Genet.

[7] Geoffriau E, Kahane R, Rancillac M (1997). Variation of gynogenesis ability in onion (*Allium cepa* L.). Euphytica.

[8] Cardoso JC, Teixeira da Silva JA (2013). Gerbera micropropagation. Biotechnol Adv.

[9] Gleddie S, Keller W, Setterfield G (1983). Somatic embryogenesis and plant regeneration from leaf explants and cell suspensions of *Solanum melongena* (eggplant). Can J Bot.

[10] Sharma P, Rajam MV (1995). Genotype, explant and position effects on organogenesis and somatic embryogenesis in eggplant ( *Solanum melongena* L.). J Exp Bot.

[11] Franklin G, Sheeba CJ, Lakshmi SG (2004). Regeneration of eggplant (*Solanum melongena* L.) from root explants. Vitr Cell Dev Biol – Plant.

[12] Taher D, Solberg S, Prohens J, Chou Y, Rakha M, Wu T (2017). World vegetable center eggplant collection: origin, composition, seed dissemination and utilization in breeding. Front Plant Sci.

[13] Altpeter F, Springer NM, Bartley LE, Blechl AE, Brutnell TP, Citovsky V, Conrad LJ, Gelvin SB, Jackson DP, Kausch AP, Lemaux PG, Medford JI, Orozco-Cárdenas ML, Tricoli DM, Van Eck J, Voytas DF, Walbot V, Wang K, Zhang ZJ, Stewart CN (2016). Advancing crop transformation in the era of genome editing. Plant Cell.

[14] Haque E, Taniguchi H, Hassan MM, Bhowmik P, Karim MR, Śmiech M, Zhao K, Rahman M, Islam T (2018). Application of CRISPR/Cas9 genome editing technology for the improvement of crops cultivated in tropical climates: recent progress, prospects, and challenges. Front Plant Sci.

[15] Limera C, Sabbadini S, Sweet JB, Mezzetti B (2017). New biotechnological tools for the genetic improvement of major woody fruit species. Front Plant Sci.

[16] Gilissen LJW, van Staveren MJ, Creemers-Molenaar J, Verhoeven HA (1993). Development of polysomaty in seedlings and plants of *Cucumis sativus* L. Plant Sci.

[17] Smulders MJM, Rus-Kortekaas W, Gilissen LJW (1994). Development of polysomaty during differentiation in diploid and tetraploid tomato (*Lycopersicon esculentum*) plants. Plant Sci.

[18] Mishiba KI, Mii M (2000). Polysomaty analysis in diploid and tetraploid *Portulaca grandiflora*. Plant Sci.

[19] Meric C, Dane F (2005). Determination of ploidy levels in *Ipheion uniflorum* (R. C. Graham) Rafin (Liliaceae). Acta Biol Hung.

[20] Letham DS (1966). Purification and probable identity of a new cytokinin in sweet corn extracts. Life Sci.

[21] Narasimhulu SB, Kirti PB, Prakash S, Chopra VL (1993). Rapid and high frequency shoot regeneration from hypocotyl protoplasts of *Brassica nigra*. Plant Cell Tissue Organ Cult.

[22] Bhadra SK, Hammatt N, Power JB, Davey MR (1994). A reproducible procedure for plant regeneration from seedling hypocotyl protoplasts of *Vigna sublobata* L. Plant Cell Rep.

[23] Hossain M, Imanishi S, Egashira H (1995). An improvement of tomato protoplast culture for rapid plant regeneration. PCTOC.

[24] Yadav NR, Sticklen MB (1995). Direct and efficient plant regeneration from leaf explants of *Solanum tuberosum* l. cv. Bintje. Plant Cell Rep.

[25] Chen L, Adachi T (1994). Plant regeneration via somatic embryogenesis from cotyledon protoplast of tomato (*Lycopersicon esculentum* Mill.). Breed Sci.

[26] Richwine AM, Tipton JL, Thompson GA (1995). Establishment of aloe, gasteria, and haworthia shoot cultures from inflorescence explants. HortScience.

[27] Rolli E, Brunoni F, Bruni R (2016). An optimized method for in vitro propagation of african baobab (*Adansonia digitata* L.) using two-node segments. Plant Biosyst.

[28] Farooq QUA, Fatima A, Murtaza N, Hussain FF. In vitro propagation of olive cultivars ‘Frontio’, ‘Earlik’, ‘Gemlik’. Acta Hortic. 2017:249–56. 10.17660/ActaHortic.2017.1152.34.

[29] Singh AK, Verma SS, Bansal KC (2010). Plastid transformation in eggplant (*Solanum melongena* L.). Transgenic Res.

[30] Muktadir MA, Habib MA, Khaleque Mian MA, Yousuf Akhond MA (2016). Regeneration efficiency based on genotype, culture condition and growth regulators of eggplant (*Solanum melongena* L.). Agric Nat Resour.

[31] Rotino GL (1996). Haploidy in eggplant.

[32] Emrani Dehkehan M, Moieni A, Movahedi Z (2017). Effects of zeatin riboside, mannitol and heat stress on eggplantn (*Solanum melongena* L.) anther culture. Imam Khomeini Int Univ Biotechnol Soc.

[33] Magioli C, de Oliveira DE, Rocha APM, Mansur E (1998). Efficient shoot organogenesis of eggplant ( *Solanum melongena* L.) induced by thidiazuron. Plant Cell Rep.

[34] Scoccianti V, Sgarbi E, Fraternale D, Biondi S (2000). Organogenesis from *Solanum melongena* l. (eggplant) cotyledon explants is associated with hormone-modulated enhancement of polyamine biosynthesis and conjugation. Protoplasma.

[35] Rahman M, Asaduzzaman M, Nahar N, Bari M (2006). Efficient plant regeneration from cotyledon and midrib derived callus in eggplant (*Solanum melongena* L.). J Bio-Science.

[36] Bhat SV, Jadhav A, Pawar BD, Kale AA, Chimote V, Pawar SV (2013). In vitro shoot organogenesis and plantlet regeneration in brinjal (*Solanum melongena* L.). N Save Nat to Surviv.

[37] Swathy PS, Rupal G, Prabhu V, Mahato KK, Muthusamy A (2017). In vitro culture responses, callus growth and organogenetic potential of brinjal (*Solanum melongena* L.) to he-ne laser irradiation. J Photochem Photobiol B Biol.

[38] Acquadro A, Barchi L, Gramazio P, Portis E, Vilanova S, Comino C (2017). Coding SNPs analysis highlights genetic relationships and evolution pattern in eggplant complexes. PLoS One.

[39] Ranil RHG, Prohens J, Aubriot X, Niran HML, Plazas M, Fonseka RM, Vilanova S, Fonseka HH, Gramazio P, Knapp S (2017). *Solanum insanum* L. (subgenus *Leptostemonum* bitter, Solanaceae), the neglected wild progenitor of eggplant (*S. melongena* L.): a review of taxonomy, characteristics and uses aimed at its enhancement for improved eggplant breeding. Genet Resour Crop Evol.

[40] Souza Fernanda Vidigal Duarte, Garcia-Sogo Begoña, Souza Antonio da Silva, San-Juán Amparo Pérez, Moreno Vicente (2006). Morphogenetic response of cotyledon and leaf explants of melon (Cucumis melo L.) cv. Amarillo Oro. Brazilian Archives of Biology and Technology.

[41] Abdalmajid M, Mohd RI, Mihdzar AK, Halimi MS (2011). In vitro performances of hypocotyl and cotyledon explants of tomato cultivars under sodium chloride stress. Afr J Biotechnol.

[42] Matand K, Wu N, Wu H, Tucker E, Love K (2013). More improved peanut (*Arachis hypogaea* L.) protocol for direct shoot organogenesis in mature dry-cotyledonary and root tissues. J Biotech Res.

[43] Pierik RLM. In vitro culture of higher plants. Dordrecht: Kluwer Academic Publishers; 1997.

[44] Waman AA, Bohra P, Sathyanarayana BN, Umesha K, Mukunda GK, Ashok TH, Gowda B (2015). Optimization of factors affecting in vitro establishment, ex vitro rooting and hardening for commercial scale multiplication of silk banana (*Musa* aab). Erwerbs-Obstbau.

[45] Sarker R, Yesmin S, Hoque M (2006). Multiple shoot formation in eggplant (*Solanum melongena* L.). Plant Tissue Cult Biotechnol.

[46] Van Den Bulk RW, Lgffler HJM, Lindhout WH, Koornneef M (1990). Somaclonal variation in tomato: effect of explant source and a comparison with chemical mutagenesis. Theor Appl Genet.

[47] Chen W, Tang CY, Kao YL (2009). Ploidy doubling by in vitro culture of excised protocorms or protocorm-like bodies in *Phalaenopsis species*. Plant Cell Tissue Organ Cult.

[48] Syfert MM, Castaneda-Alvarez NP, Khoury CK, Sarkinen T, Sosa CC, Achicanoy HA, Bernau V, Prohens J, Daunay MC, Knapp S (2016). Crop wild relatives of the brinjal eggplant (*Solanum melongena*): Poorly represented in genebanks and many species at risk of extinction. Am J Bot.

[49] Muñoz-Falcón JE, Prohens J, Vilanova S, Nuez F (2009). Diversity in commercial varieties and landraces of black eggplants and implications for broadening the breeders’ gene pool. Ann Appl Biol.

[50] Kaushik P, Prohens J, Vilanova S, Gramazio P, Plazas M (2016). Phenotyping of eggplant wild relatives and interspecific hybrids with conventional and phenomics descriptors provides insight for their potential utilization in breeding. Front Plant Sci.

[51] Plazas M, Vilanova S, Gramazio P, Rodriguez-Burruezo A, Rajakapasha R, Ramya F, Niran L, Fonseka H, Kouassi B, Kouassi A, Kouassi A, Prohens J (2016). Interspecific hybridization between eggplant and wild relatives from different genepools. J Am Soc Hortic Sci.

[52] Kouassi B, Prohens J, Gramazio P, Kouassi AB, Vilanova S, Galán-Ávila A, Herraiz FJ, Kouassi A, Seguí-Simarro JM, Plazas M (2016). Development of backcross generations and new interspecific hybrid combinations for introgression breeding in eggplant (*Solanum melongena*). Sci Hortic (Amsterdam).

[53] García-Fortea E, Gramazio P, Vilanova S, Fita A, Mangino G, Villanueva G, Arrones A, Knapp S, Prohens J, Plazas M (2019). First successful backcrossing towards eggplant (*Solanum melongena* ) of a New World species, the silverleaf nightshade (*S. elaeagnifolium* ), and characterization of interspecific hybrids and backcrosses. Sci Hortic.

[54] Murashige T, Skoog F (1962). A revised medium for rapid growth and bio agsays with tobacco tissue cultures. Physiol Plant.

[55] Dpooležel J, Binarová P, Lcretti S (1989). Analysis of nuclear DNA content in plant cells by flow cytometry. Biol Plant.

[56] Ihaka R, Gentleman R (1996). R: a language for data analysis and graphics. J Comput Graph Stat.

